# Functional Impact of *ABCB1* Variants on Interactions between P-Glycoprotein and Methadone

**DOI:** 10.1371/journal.pone.0059419

**Published:** 2013-03-19

**Authors:** Chin-Chuan Hung, Mu-Han Chiou, Yu-Ning Teng, Yow-Wen Hsieh, Chieh-Liang Huang, Hsien-Yuan Lane

**Affiliations:** 1 Department of Pharmacy, College of Pharmacy, China Medical University, Taichung, Taiwan; 2 Department of Pharmacy, China Medical University Hospital, Taichung, Taiwan; 3 Department of Pharmacy, Cathay General Hospital, Taipei, Taiwan; 4 Department of Psychiatry, China Medical University Hospital, Taichung, Taiwan; 5 Institute of Clinical Medical Science, College of Medicine, China Medical University, Taichung, Taiwan; Universidade Federal do Rio de Janeiro, Brazil

## Abstract

Methadone is a widely used substitution therapy for opioid addiction. Large inter-individual variability has been observed in methadone maintenance dosages and P-glycoprotein (P-gp) was considered to be one of the major contributors. To investigate the mechanism of P-gp’s interaction with methadone, as well as the effect of genetic variants on the interaction, Flp-In™-293 cells stably transfected with various genotypes of human P-gp were established in the present study. The RNA and protein expression levels of human P-gp were confirmed by real-time quantitative RT-PCR and western blot, respectively. Utilizing rhodamine 123 efflux assay and calcein-AM uptake study, methadone was demonstrated to be an inhibitor of wild-type human P-gp via non-competitive kinetic (IC_50_ = 2.17±0.10 µM), while the variant-type human P-gp, P-gp with 1236T-2677T-3435T genotype and P-gp with 1236T-2677A-3435T genotype, showed less inhibition potency (IC_50_ = 2.97±0.09 µM and 4.43±1.10 µM, respectively) via uncompetitive kinetics. Methadone also stimulated P-gp ATPase and inhibited verapamil-stimulated P-gp ATPase activity under therapeutic concentrations. These results may provide a possible explanation for higher methadone dosage requirements in patients carrying variant-type of P-gp and revealed the possible drug-drug interactions in patients who receive concomitant drugs which are also P-gp substrates.

## Introduction

Opioid addiction is a severe mental disorder, of which chronic and relapsing course is also a heavy burden of patients and their families and even societies [1]. Methadone maintenance therapy is widely used for opioid addiction [2]–[4]. Large inter-individual variability of methadone treatment, carrying a great challenge in clinical practice is related to metabolizing enzymes, drug targets, and drug transporters [5], [6]. Among the efflux drug transports expressed in human, P-glycoprotein (P-gp) is one of the major transporters expressed at the human blood-brain barrier and recent studies have suggested that it may contribute to the inter-individual variability of dosage requirement of methadone maintenance therapy [7]–[11].

Methadone has been demonstrated to be a substrate of P-gp [12]–[14] and pharmacokinetics of methadone in rats and humans were found to be altered by P-gp [15]–[17]. P-gp mediated transport of methadone significantly affected the pharmacologic response to methadone, such as analgesic effect [18] and inhibition of P-gp was shown to enhance the intestinal absorption of methadone [15]. P-gp is a member of the ATP-binding cassette (ABC) superfamily and functions as an efflux pump which transports substrates from intracellular to extracellular [19], [20]. It is expressed abundantly in tissues with biological barrier functions, such as the epithelia of the liver, kidney, intestine and the endothelial cell of blood-brain barrier (BBB) [21]. It has a broad range of substrates and many therapeutic agents are substrates of P-gp [20], [22]–[25], thus the function of P-gp may influence the pharmacokinetics of drugs and further affect treatment outcomes.

P-gp is encoded by the human *ABCB1* gene, which is a highly polymorphic gene with more than 50 single nucleotide polymorphisms (SNPs) identified in the NCBI database. The most frequently studied SNPs in *ABCB1* gene were c.1236C>T (rs1128503), c.2677G>T/A (rs2032582) and c.3435C>T (rs1045642). It has been demonstrated that these SNPs may be associated with P-gp expression, drug responses and disease susceptibility [26]–[28]. The effect of *ABCB1* genetic polymorphisms on methadone maintenance therapy has been studied and inconsistent results were reported [7]–[9]. Several reasons may account for the discrepancy functional significance, including analyses of single SNPs rather than haplotypes, ethnic-specific allele frequencies [27], [29] and the effect of other transporters or metabolizing enzymes. Since these three SNPs are reported to be in strong linkage disequilibrium [30], [31], haplotypes may provide more consistent results. Recently, two studies from different groups provided consistent results that patients with variant genotype combination, such as TT-TT-TT, tended to require higher methadone maintenance doses [10], [11]. Moreover, drug addicts may concomitantly use antiretroviral agents, protease inhibitors and antipsychotics that may also be P-gp substrates [23], [32]–[34]. These drug-transporter interactions may lead to significant drug-drug interactions in clinical practice. Till now, the molecular mechanism of interaction between methadone and P-gp remains veiled. Moreover, how the polymorphisms of the *ABCB1* gene influence this interaction requires elucidation.

In order to investigate the functional effect of *ABCB1* haplotype combinations on methadone-P-gp interactions, we established cell lines carrying human P-gp with haplotype combinations of *ABCB1* c.1236C>T, c.2677G>T/A, and c.3435C>T. By this reproducible and validated model, the underlying mechanism of methadone-P-gp interaction and the functional effects of *ABCB1*’s genetic polymorphisms were studied.

## Materials and Methods

### Materials

Rhodamine 123, calcein-AM, R-(+)-Verapamil and racemic methadone were purchased from Sigma Chemical Co (St. Louis, MO, U.S.A.). The Flp-In ™ system, Flp-In™-293 cells (human embryonic kidney cells), zeocin, hygromycin B and all cell culture medium and reagents were obtained from Invitrogen (Carlsbad, CA, USA). Human *ABCB1* cDNA in pMDRA1 was provided by the Riken BRC DNA bank (RDB No. 1372) (Ibaraki, Japan). All restriction enzymes were purchased from New England Biolabs (Ipswich, MA, USA).

### Construction of Expression Plasmids and Site-directed Mutagenesis

Plasmid pMDRA1 containing a full-length human *ABCB1* cDNA was digested with Sac I and Xho I, and the *ABCB1* cDNA fragment was ligated into pET21a(+) which was pre-cut with Sac I and Xho I. The recombinant plasmid was then digested with Bam HI and Xho I, and the insert was purified by 0.8% agarose gel and further ligated into the mammalian expression vector pcDNA5/FRT pre-cut with Bam HI and Xho I (pcDNA5/*ABCB1*). The QuickChange™ Site-Directed Mutagenesis Kit (Stratagene, La Jolla, CA, USA) was used to introduce point mutations (1236C>T, 2677G>T, 2677G>A, 3435C>T) into the pcDNA5/*ABCB1* according to the instructions. Triple mutations (1236T-2677T-3435T and 1236T-2677A-3435T) were created to evaluate the effect of these genotypes on human P-gp function. The sequences were confirmed by direct sequencing.

### Cell Line Establishment

Flp-In™-293 cells were cultured in DMEM medium supplemented with 10% fetal bovine serum and selected with 100 µg/mL zeocin at 37°C, 95% humidity and 5% CO_2_. The pcDNA5/*ABCB1* containing different genotypes of *ABCB1* cDNA and pOG44 (the Flp recombinase expression plasmid) were co-transfected into the Flp-In™-293 cells. Stable cell lines were selected on the basis of hygromycin B resistance. The expressions of Pgp were confirmed by Western blot analysis and the genotypes of the transfected cells were confirmed by sequencing.

### Immunoblot Analysis

Membrane proteins (2 µg) were separated by sodium dodecyl sulphate-polyacrylamide (10%) gel electrophoresis and transferred to PVDF membrane (Millipore, Billerica, MA, USA) using a Transblotter™ (Bio-Rad, Hercules, CA, USA). After blocking by 5% (w/v) nonfat powdered milk, the membrane was incubated with the anti-P-gp monoclonal antibody C219 (ALEXIS Biochemicals, Lausen, Switzerland) at 1∶500 dilution in Tris-buffered saline supplemented with 0.1% Tween 20 (T-TBS) at 4°C over night, washed four times in T-TBS and incubated with goat anti-mouse IgG at room temperature for one hour. The C219 is a monoclonal antibody to P-gp and recognizes an internal, highly conserved amino acid sequence found in both protein isoforms, P-gp and MDR3 P-gp [35]. The transferred proteins were detected by enhanced chemiluminescence reagents (Amersham, Piscataway, NJ, USA) and the chemiluminescence was detected by using the UVP BioChemi Image System (Upland, CA, USA.).

### Real-time Quantitative RT-PCR

*ABCB1* mRNA levels were evaluated by real-time quantitative RT-PCR utilizing an Applied Biosystems Taqman assay using 1 µg of total RNA. Total RNA was extracted from the transfected cells by the Qiagen RNeasy kit (Valencia, CA, USA). Taqman primers and probes for human *ABCB1* and *GAPDH* were Assay on Demand reagents (Applied Biosystem, Foster City, CA, USA) and were analyzed using an ABI Prism 7900 Sequence Detection System. The relative expression of *ABCB1* mRNA was normalized to the amount of *GAPDH* in the same cDNA by using the standard curve method described by the manufacturer.

### Cytotoxicity Assay

Cytotoxicities of methadone at concentrations used in the present study were determined by MTT assay. Briefly, the cells were seeded into 24-well plates and cultured overnight. Various concentrations of methadone were then added and incubated for 48 hours. After incubation, the medium was removed and added fresh culture medium 200 µl containing 0.5 mg/ml MTT and then incubated at 37°C for 4 hours. After incubation, the medium was removed and 200 µl DMSO was added, and incubation for 30 minutes at room temperature. After incubation, 100 µl of solution was transferred to a 96-well plate and absorbance was measured using an ELISA plate reader (Molecular Devices, Sunnyvale, USA) at 570 nm with a reference wavelength of 650 nm.

### P-gp ATPase Activity Assays

The effect of methadone on P-gp ATPase activity was examined by Pgp-GIO assay system (Promega, Madison, WI, USA) according to manufacturer’s protocol. To be brief, in a 96-well untreated white plate, 25 µg recombinant human Pgp was incubated with P-gp-GIO assay buffer (untreated control) or 200 µM verapamil (positive control of drug induced P-gp ATPase activity) or 100 µM sodium orthovanadate (selective inhibitor of P-gp ATPase activity) or 1–40 µM methadone. The reaction was initiated by adding 5 mM MgATP and incubated for 40 min at 37°C. After removing the plate from 37°C heat source, the luminescence was initiated by adding 50 µl of ATP detection reagent. The luminescence was read after 20 min of incubation at room temperature on the SpectraMax Gemini XS microplate spectrofluorometer (Molecular Devices Co., Sunnyvale, CA, USA). Each experiment was performed at least three times, each in triplicate on different days.

### Rhodamine 123 Intracellular Uptake and Efflux Assays

For FACS analysis, 5×10^5^ cells were harvested after trypsinization by centrifugation and resuspended in fresh culture medium. Rhodamine123 (final concentration 1 µM) was added and the cells were incubated in a water bath at 37°C in the dark. Cells were pelleted at 1, 5, 10, 15, 30, 45, 60, and 90 min. After washed with ice-cold PBS, cells were resuspended in 1-ml, ice-cold PBS prior to FACS analysis using CellQuest software (Becton Dickinson, Franklin Lakes, NJ, USA). Each cell type was performed at least three experiments, each in triplicate on different days.

For efflux assays, 1×10^5^ cells were seeded on 96-well plates and cultured for 24 hours. After incubation, cells were washed with warm Hanks’ balanced salt solution (HBSS) and pre-incubated with HBSS for 30 min, and subsequently incubated in 1 µM rhodamine123 for 30 min with or without methadone in triplicate. After washed with warm PBS, cells were allowed to efflux rhodamine123 for 10 min at 37°C in the incubator. Samples (150 µl) were collected and transferred to 96-well black plates. Rhodamine123 concentrations were measured using a set of standards (from 62.5 nM to 8 µM) in HBSS analyzed by the SpectraMax Gemini XS microplate spectrofluorometer (Molecular Devices Co., Sunnyvale, CA, USA) with the excitation set at 485 nm and the emission set at 535 nm. The measured rhodamine123 concentrations were normalized to the total protein content in the cells. Total protein concentrations were measured by a microplate assay protocol (Dc protein assay reagent, Bio-Rad, Hercules, CA, USA), and bovine serum albumin was used as the standard. Each experiment was repeated at least three times across cell passages to ensure stability of transfection and consistency and reproducibility of the experiments.

### Calcein-AM Uptake Assay

For calcein-AM uptake study, 1×10^5^ cells were plated on 96-well black plates and cultured for 24 hours. Before the uptake assay, cells were washed with warm Hanks’ balanced salt solution (HBSS) and pre-incubated with HBSS for 30 min, and subsequently with methadone for 30 min in triplicate. After pre-incubation, calcein AM (final 1 µM) was added and incubated at 37°C in the incubator for 30 min. After washed by ice-cold HBSS, cells were lysed with 1% Triton X-100 and calcein fluorescence generated within the cells was analyzed by the SpectraMax Gemini XS microplate spectrofluorometer (Molecular Devices Co., Sunnyvale, CA, USA) with the excitation set at 485 nm and the emission set at 535 nm. Each experiment was performed at least three times, each in triplicate on different days.

### Data Analysis

For ATPase Assay data analysis, basal P-gp activity, verapamil-stimulated P-gp ATPase activity, methadone effect on P-gp ATPase activity and fold stimulation by methadone were calculated as following equations:

Basal P-gp activity:
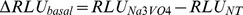



Verapamil-stimulated P-gp ATPase activity:
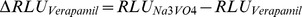



Methadone effect on P-gp ATPase activity:
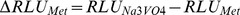



Fold stimulation by methadone:
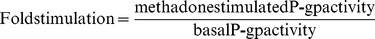



For evaluation of inhibitor potency, IC_50_ values were calculated by the following equation:
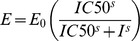
Where E is the observed efflux in the presence of inhibitor; E_0_, the efflux in the absence of inhibitor; I, the inhibitor concentration; IC50, the concentration that caused 50% inhibition of the maximal drug effect and s is the slope factor.

For kinetic studies, kinetic parameters were estimated by nonlinear regression using Scientist v2.01 (MicroMath Scientific Software, Salt Lake City, UT, U.S.A.) according to the following equation:
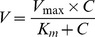
Where V denoted the efflux rate; V_max_, the maximal efflux rate; Km, the Michaelis-Menten constant and C is the substrate concentration.

Statistical comparisons among different cells were performed by using SYSTAT v10 (Systat, Inc., Evanston, IL, U.S.A.). Statistical differences were evaluated by ANOVA and post hoc analysis (Tukey’s test) and the statistical significance was set at p value <0.05.

## Results

### Expression of Pgp in Established Cell Lines

**Figure 1:**
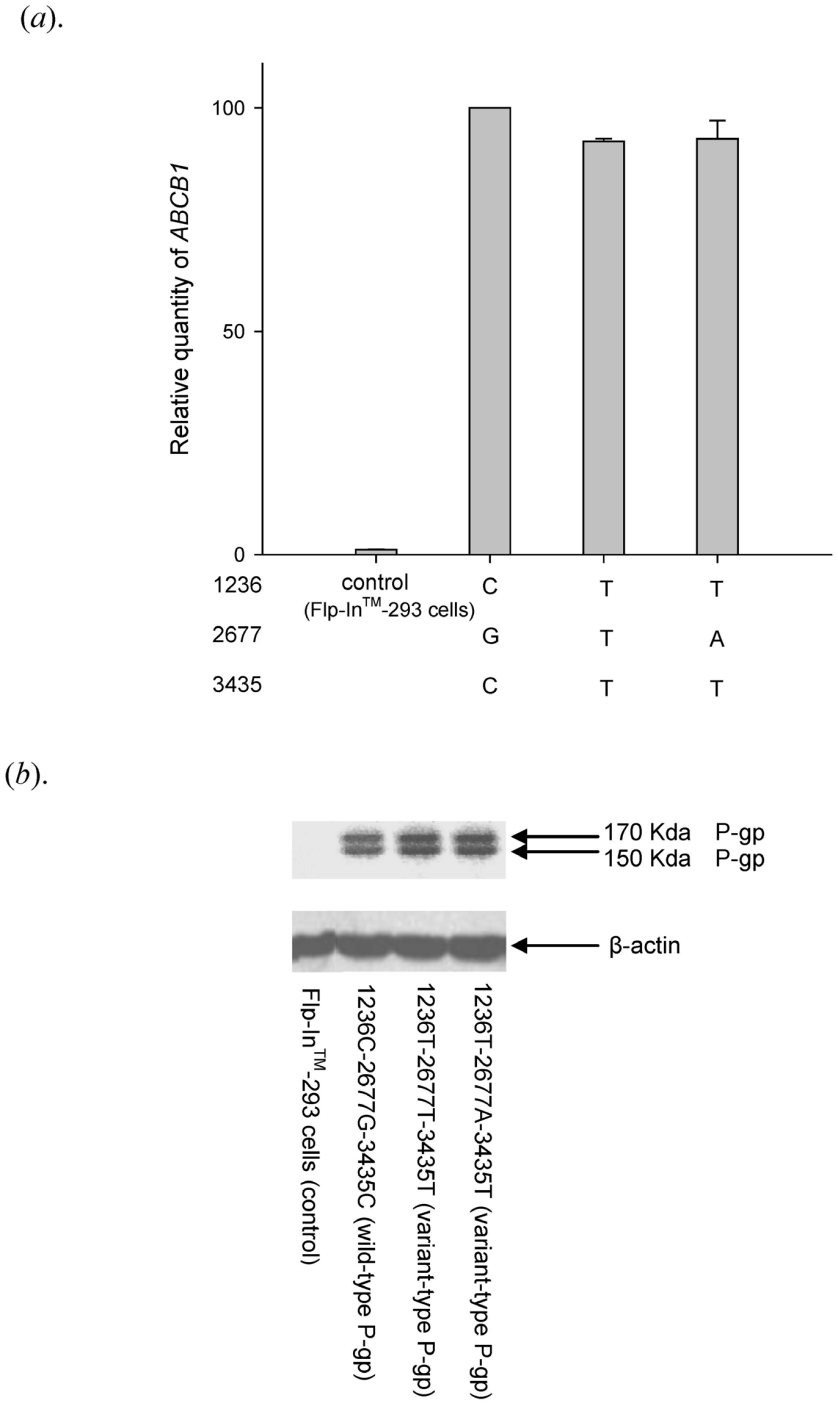
RNA and protein expression of human P-gp in control cells (Flp-In™-293 cells), cells expressed wild-type P-gp and cells expressed variant-type P-gp. (*a*) RNA expression was detected by real-time quantitative RT-PCR. There was no significant different RNA expression among cells expressed wild-type P-gp and cells expressed variant-type P-gp, whereas little *ABCB1* gene expression was detected in the control cells. Data were presented as mean ± SE of at least three experiments, each in triplicate.(*b*) Western blot of P-gp expression with C219 monoclonal antibody (2 µg protein/lane). Arrows marked the mature fully glycosylated (∼170 kD) and immature P-gp (∼150 kD). The expression of β-actin was used as the loading control. No significant difference was detected among cells expressed wild-type P-gp and cells expressed variant-type P-gp.

There was no significant difference among the mRNA and protein expression levels of wild type *ABCB1* and the two variant types, and little expression of *ABCB1* mRNA and protein was found in the Flp-In™-293 cells (Fig. 1). Methadone at concentrations ranging from 0.5 to 40 µM did not affect Flp-In™-293 cells viability using MTT assay.

### Functional Evaluation of P-gp

**Figure 2:**
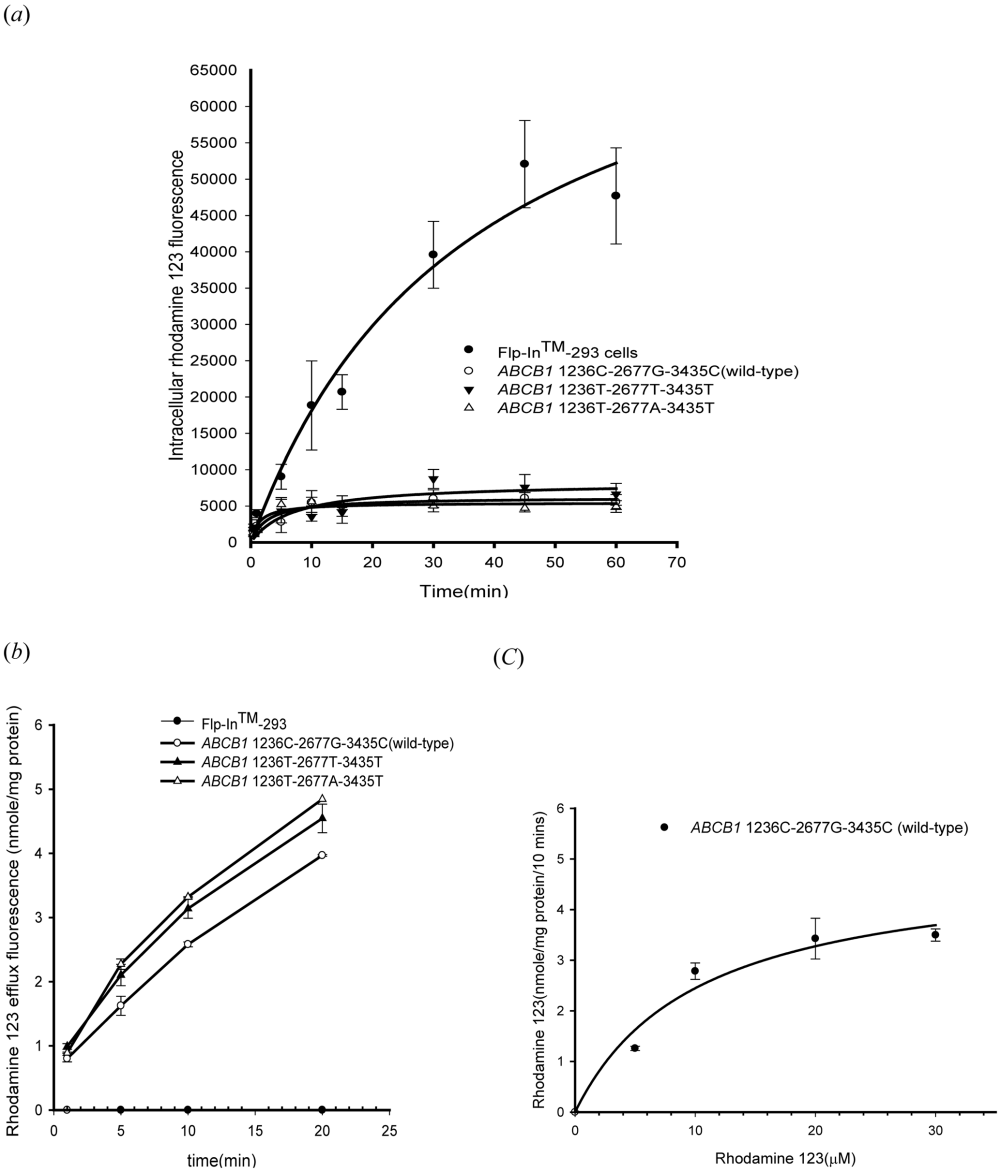
Validation of the function of established cell lines by rhodamine 123. (*a*) Time curve of 1 µM rhodamine 123 intracellular accumulation. The intracellular accumulation of rhodamine 123 in human P-gp expressed cells was saturated after 30 minutes; therefore, the following experiments were performed after 30 minutes of incubation with rhodamine 123. (*b*) Time curve of rhodamine 123 efflux transport. After incubation with 1 µM rhodamine 123, cells were allowed to efflux rhodamine 123 at different time points. The initial rate of rhodamine 123 efflux was 10 minutes. (*c*) Concentration-dependent efflux of rhodamine 123 in wild-type P-gp. Efflux of rhodamine 123 followed Michaelis-Menten kinetics in the concentration range of0.5–30 µM. Data were presented as mean ± SE of at least three experiments, each in triplicate.

Rhodamine123, a well-known P-gp substrate, was used as a model compound to investigate the effects of methadone on P-gp function in the present study. Time course and concentration-dependent uptake and efflux of rhodamine123 by wild-type P-gp are shown in figure 2. Total efflux of rhodamine 123 was a combination of a saturable process and first-order non-specific transport. The K_M_ and Vmax values for rhodamine 123 efflux in wild-type P-gp were estimated to be 16.58±3.01 µM and 6.69±0.85 nmole/mg protein/10 min, respectively, whereas the non-specific first-order rate constant was estimated to be 12.08±1.23 µL/mg protein/10 min. The K_M_ and Vmax values for rhodamine 123 efflux in wild-type and variant-type P-gp were similar (Table 1). Since the intrinsic clearance, Vmax/K_M_, of rhodamine 123 was 403.5 µL/mg protein/10 mins, non-specific transport accounted for only 2.9% of the total efflux.

**Table 1:** Effect of methadone on rhodamine123 transport in HEK293 cells expressed human wild-type P-gp and variant-type P-gp.

	Nonlinear kinetic parameters			
*ABCB1* 1236C-2677G-3435C (wild type)	Vmax (nmole/mg protein/10 min)		Km(µM)	
Nonlinear regression				
Rhodamine 123 only	6.69±0.85		16.58±3.01	
+ methadone, 2.5 µM	3.79±0.76		16.60±6.08	
+ methadone, 5 µM	3.10±0.30*		16.20±4.08	
Methadone Ki estimation				
Ki from Lineweaver–Burk(µM)^a^				2.77±0.15
IC_50_(µM)				2.17±0.10
*ABCB1*1236T-2677T-3435T				
Nonlinear regression				
Rhodamine 123 only	8.29±0.89	18.80±2.00		
+ methadone, 2.5 µM	5.02±0.27*	15.09±0.58		
+ methadone, 5 µM	3.68±0.35*	9.74±0.74*		
Methadone Ki estimation				
Ki from Lineweaver–Burk(µM)				3.92±0.07
IC_50_(µM)				2.97±0.09
*ABCB1*1236T-2677A-3435T				
Nonlinear regression				
Rhodamine 123 only	7.34±0.63	14.81±1.72		
+ methadone, 2.5 µM	3.84±0.08*	8.50±0.55*		
+ methadone, 5 µM	2.44±0.11*	7.89±0.20*		
Methadone Ki estimation				
Ki from Lineweaver–Burk(µM)				4.53±1.17
IC_50_(µM)				4.43±1.10

All results were presented as mean±SE for at least three experiments, each in triplicate.

a The Ki values were estimated by plotting the slope of the Lineweaver-Burk plot versus inhibitor concentration, and the x intercept is equal to Ki.

* p<0.05 compared with rhodamine123 transport without methadone.

### Effects of Methadone on P-gp Transport Function

**Figure 3:**
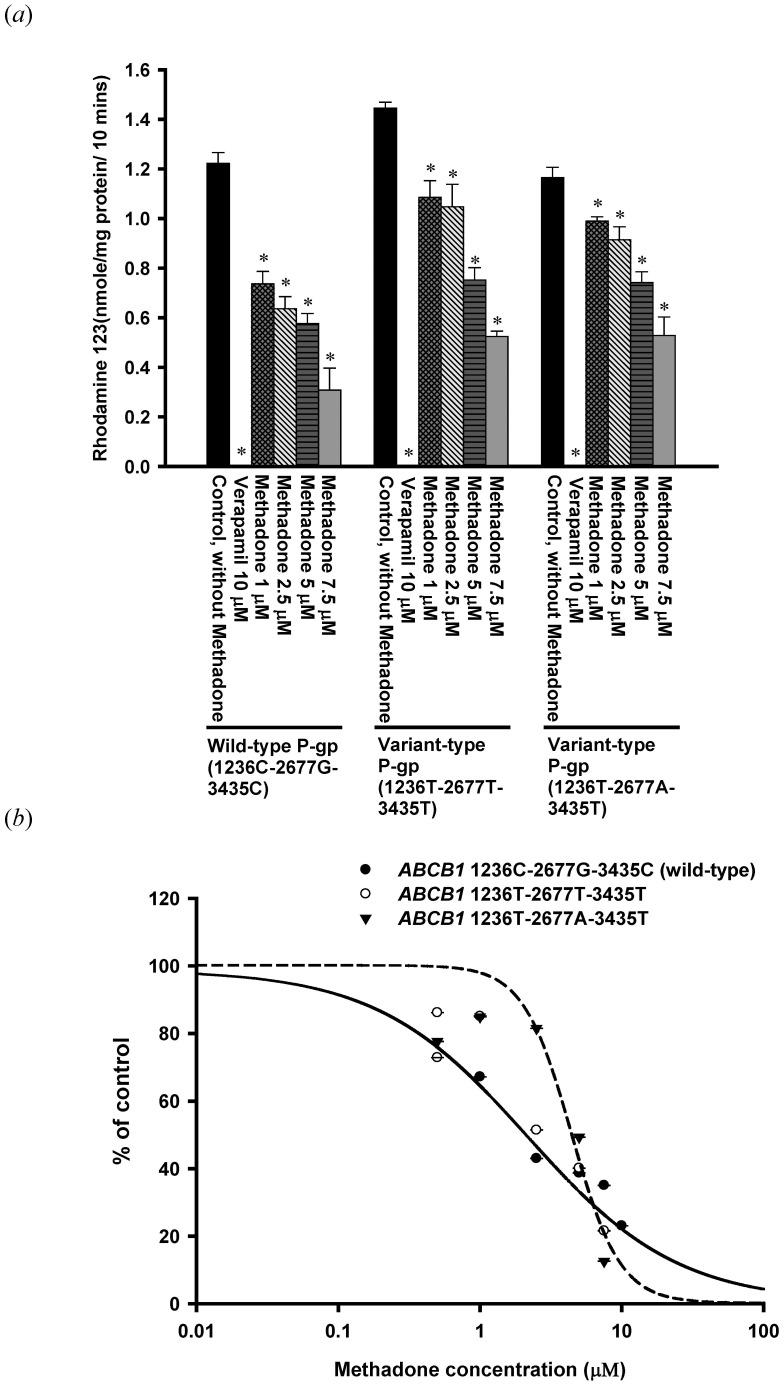
Inhibition of rhodamine 123 efflux by methadone. (*a*) Methadone inhibited P-gp mediated efflux of rhodamine 123 in a concentration dependent manner. Verapamil 10 µM was used as a positive control for the inhibition model. Methadone significantly inhibited rhodamine 123 efflux in wild-type P-gp, as well as the two variant-types of P-gp. *p<0.05 compared withrhodamine123transport without methadone. (*b*) Dose-response study of rhodamine 123 efflux in the presence of methadone (0.5–100 µM). Data were presented as mean ± SE of at least three experiments, each in triplicate.

**Figure 4:**
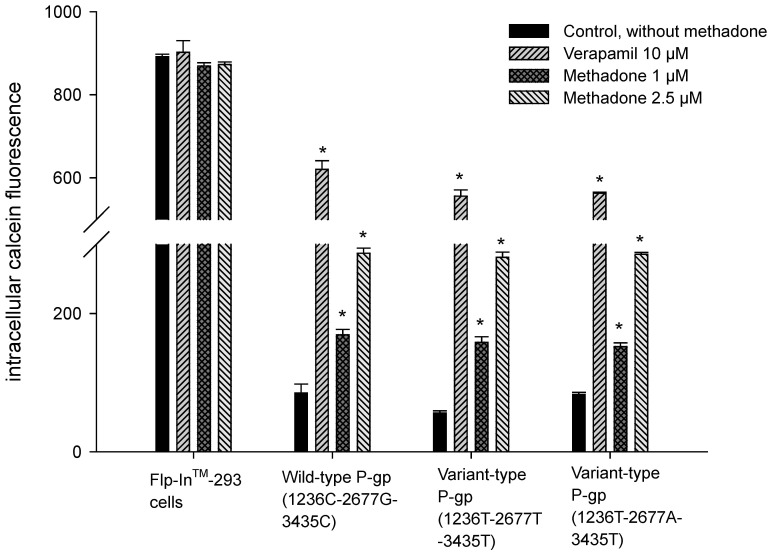
Effect of methadone on calcein-AM uptake assay. Methadone significantly increased intracellular accumulation of calcein in a dose dependent manner in wild-type P-gp, as well as the two variant-types of P-gp. Verapamil 10 µM was used as a positive control.Data were presented as mean ± SE of at least three experiments, each in triplicate. *p<0.05 compared withcalcein-AM uptake without methadone.

To determine the time interval of rhodamine 123 efflux in the inhibition study, the linear range of efflux was used. In terms of pharmacokinetics, this is the initial rate of rhodamine 123 efflux. In the present study, the linear range of rhodamine 123 efflux was the initial 10 min. Therefore, rhodamine 123 efflux at 10 min was used in the inhibition study. The efflux of rhodamine 123 was significantly inhibited by methadone in both cells expressed wild-type P-gp and variant-type P-gp (fig. 3). In the dose-response study, IC_50_ (50% inhibitory concentration) values for methadone in wild-type P-gp, P-gp with 1236T-2677T-3435T genotype, and P-gp with 1236T-2677A-3435T genotype were 2.17±0.10 µM, 2.97±0.09 µM and 4.43±1.10 µM, respectively (Table 1). The methadone inhibited P-gp transport function was further confirmed by the calcein AM uptake assay. Calcein-AM, a non-fluorescent, lipophilic compound, is also a good substrate of human P-gp. After entering cells, calcein-AM is hydrolyzed to become fluorescent. Therefore, higher intracellular calcein fluorescence indicates lower function of P-gp. Intracellular accumulation of calcein was significantly increased by methadone in a concentration-dependent manner in cells expressing wild-type P-gp and in cells expressing variant-type P-gp (fig. 4).

### Mechanism of Interaction between Methadone and P-gp

**Figure 5:**
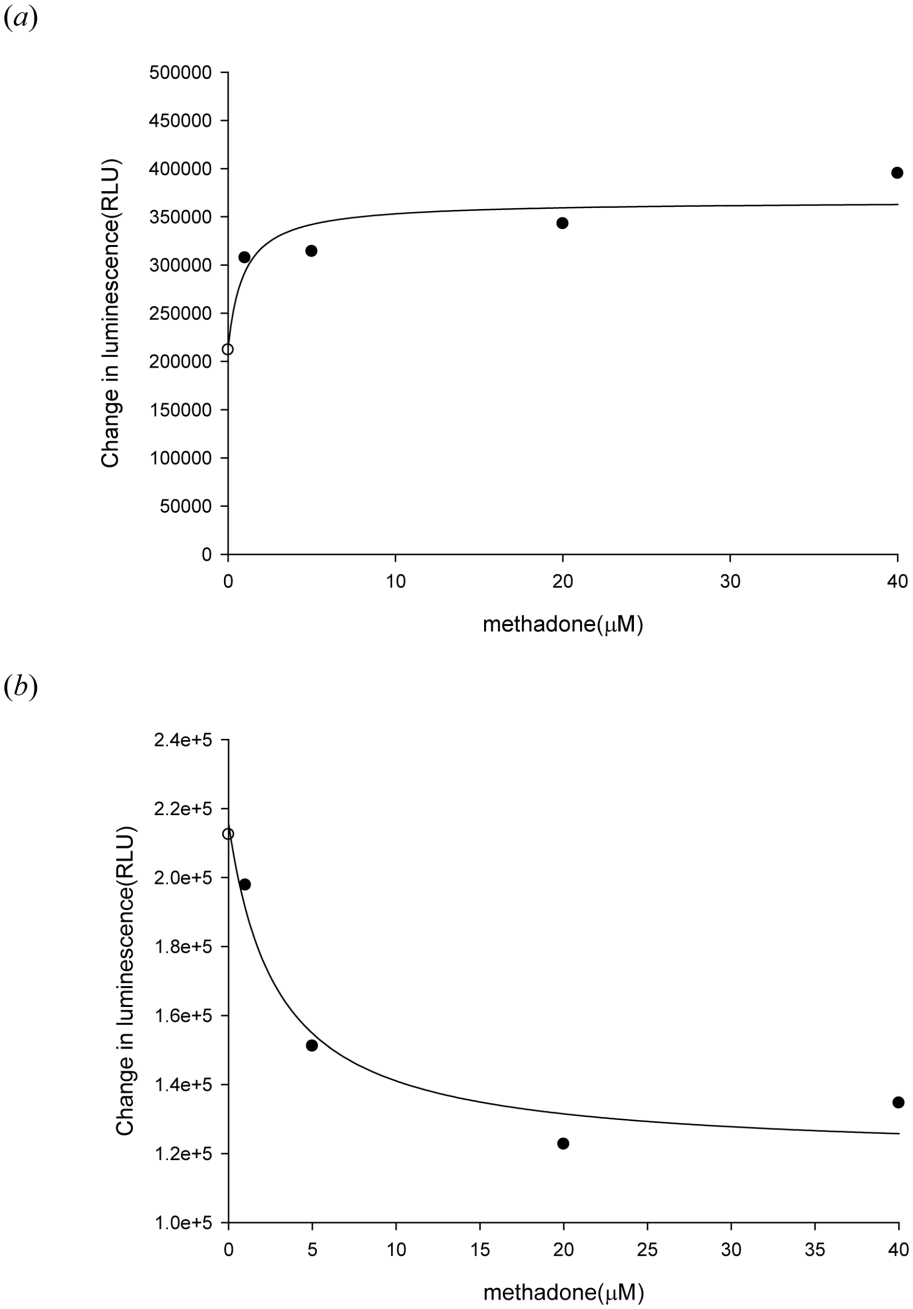
Effect of methadone on P-gp ATPase activity. (*a*) Incubation with methadone (1–40 µM) stimulated the P-gp ATPase activity. Data was analyzed in terms of RLUs. (*b*) Methadone (1–40 µM) was tested for its capacity to inhibit 200 µM verapamil-stimulated P-gp ATPase activity. Methadone stimulated the P-gp ATPase activity and inhibited 200 µM verapamil-stimulated P-gp ATPase activity at 5 µM.

To evaluate the effect of methadone on the P-gp ATPase activity, various concentrations of methadone (1–40 µM) were incubated with recombinant human P-gp. Methadone was demonstrated to stimulate P-gp ATPase activity by 1.5 fold higher than basal ATPase activity and inhibited 200 µM verapamil-stimulated P-gp ATPase activity (fig. 5).

**Figure 6:**
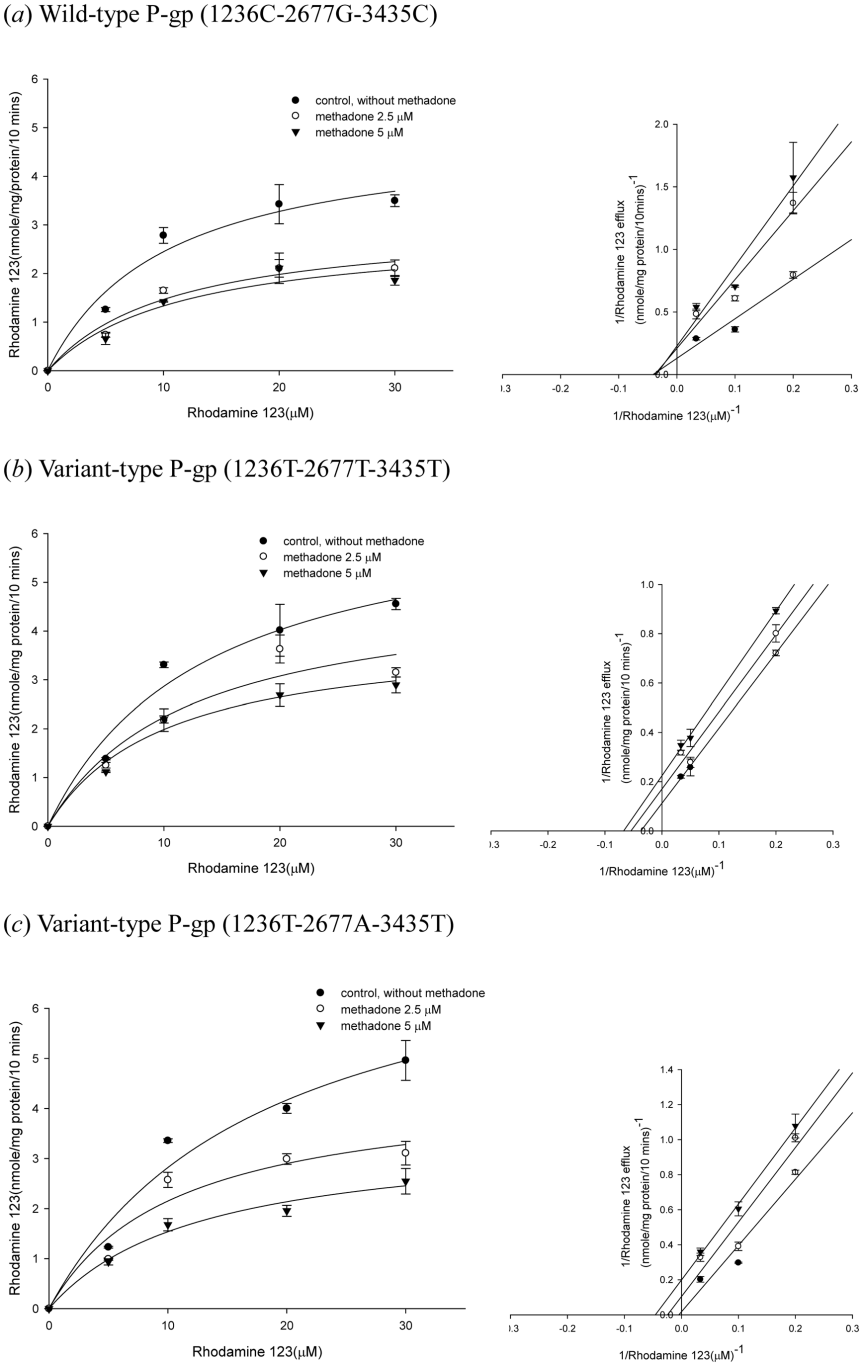
Concentration-dependent rhodamine 123 efflux (5–30 µM) in the presence or absence of methadone. The left panels showed the nonlinear regression analysis of rhodamine 123 efflux and the right panels demonstrated the Lineweaver-Burk plot analysis of rhodamine 123 efflux. (*a*) Wild-type P-gp(1236C-2677G-3435C). (*b*) Variant-type P-gp (1236T-2677T-3435T). (*c*) Variant-type P-gp (1236T-2677A-3435T). Data were presented as mean ± SE of at least three experiments, each in triplicate.

The inhibition kinetics of methadone was further investigated in the wild type and the two variant types of P-gp by Lineweaver-Burk plot (fig. 6). In the wild-type P-gp, when the concentration of methadone increased, the maximum rate of rhodamine 123 efflux (Vmax) decreased with affinity (Km) remaining unchanged (fig. 6; Table 1). These results suggested that methadone may inhibit wild-type P-gp via non-competitive inhibition. As for the two variant types of P-gp (1236T-2677T-3435T and 1236T-2677A-3435T), both Vmax and Km of rhodamine 123 decreased as the concentration of methadone increased (fig. 6; Table 1). These results indicated that methadone may inhibit P-gp carrying 1236T-2677T-3435T genotype and P-gp carrying 1236T-2677A-3435T genotype via uncompetitive inhibition.

## Discussion

P-gp, is one of the major transporters expressed in human physiological barriers, such as intestine, kidney and BBB. It has been demonstrated that inhibition of efflux transporters may cause clinically relevant drug-drug interactions and lead to insufficient treatment or adverse drug reactions [36]–[38]. The present study showed that methadone may inhibit P-gp efflux function via non-competitive inhibition and stimulate P-gp ATPase activity. Furthermore, the common genetic polymorphisms in human *ABCB1* gene, c.1236C>T, c.2677G>T/A and c.3435C>T, were demonstrated to influence not only the kinetics of interaction between methadone and P-gp but also the P-gp inhibition potency of methadone.

Several studies have evaluated the interaction between methadone and P-gp [13], [14], [39], [40]. In a rodent study, the brain concentrations of methadone in Mdr1a^−/−^ mice were 15- to 23-fold higher than in Mdr1a^+/+^ mice, indicating that methadone is a good substrate of murine P-gp [13], [40]. The analgesic effect of methadone was also found to be altered by P-gp mediated transport [17], [18]. The effect of P-gp efflux transport on pharmacokinetic of methadone was widely studied and both the absorption and distribution phases were shown to be affected in rats and humans [12], [15]–[17], [41]. The intestinal absorption of methadone was higher in the presence of verapamil [41]. Recent studies also confirmed that the P-gp mediated transport may influence the pharmacokinetics and pharmacodynamics of methadone and methadone is both a substrate and an inhibitor of human P-gp [14], [39]. However, none of these studies has explored the mechanism of methadone-P-gp interaction. The present study suggests that methadone is a non-competitive inhibitor of human wild-type P-gp and a P-gp ATPase stimulator under methadone therapeutic range (1–3 µM) [42]–[44]. The tested methadone concentrations used in the inhibition and kinetic assays were 1–7.5 µM in the present study. The concentrations of methadone reaching from 1–40 µM were only used in the ATPase assay to reach the plateau effect. The results from the inhibition assays demonstrated that methadone may inhibit wild-type human P-gp (IC50 = 2.17±0.10 µM), as well as the variant-type human P-gp, P-gp with 1236T-2677T-3435T genotype and P-gp with 1236T-2677A-3435T genotype (IC50 = 2.97±0.09 µM and 4.43±1.10 µM, respectively). For wild-type and variant-type human P-gp, 1 µM methadone was shown to significantly inhibited the efflux of rhodamine 123 and calcein AM (fig. 3(a) and fig 4.). The therapeutic rang of methadone was reported to be 1–3 µM, therefore, the effect of methadone on P-gp efflux function could be clinical relevant, especially in patients under both methadone maintenance therapy and HAART therapy. Moreover, methadone was shown to be capable of inhibiting verapamil-stimulated P-gp ATPase activity. Therefore, the interaction between methadone and human P-gp may lead to drug-drug interactions in patients receiving concomitant medications which are also P-gp substrates.

In terms of the effect of *ABCB1* genetic polymorphisms on the interaction between methadone and P-gp, c.1236C>T, c.2677G>T/A and c.3435C>T were demonstrated to be associated with methadone plasma concentrations and maximum maintenance doses [7]–[11], [45]. In Caucasian populations, subjects carrying 3435TT genotypes were found to be associated with lower methadone plasma concentrations [8], however, inconsistent result was reported regarding single locus analysis of *ABCB1* c.3435C>T [45]. The haplotype combinations of c.1236C>T, c.2677G>T/A and c.3435C>T were also associated with maximum maintenance doses of methadone [10], [11]. Patients with 1236TT-2677TT-3435TT haplotype combination tended to require higher methadone maintenance doses in Caucasians [11] while patients with 1236T-2677T-3435C or 1236T-2677G-3435T haplotypes were more likely to use higher methadone doses than wild-type carriers in Chinese [10]. The present study suggests that methadone is a human P-gp inhibitor, and that the kinetics of P-gp inhibition by methadone in P-gp carrying 1236T-2677T-3435T and P-gp carrying 1236T-2677A-3435T is uncompetitive inhibition, whereas the kinetics in wild-type P-gp is non-competitive inhibition. These variant types of P-gp were also significantly inhibited by methadone. As compared to the wild-type P-gp, methadone was a less potent inhibitor for P-gp carrying 1236T-2677T-3435T and P-gp carrying 1236T-2677A-3435T. The IC_50_ values for wild-type P-gp, P-gp carrying 1236T-2677T-3435T and P-gp carrying 1236T-2677A-3435T were 2.17 µM, 2.97 µM and 4.43 µM, respectively. Our results provide a possible explanation for the higher methadone maintenance dose in patients with variant genotypes of P-gp. It is possible that less P-gp may be inhibited in patients with variant genotypes of P-gp; therefore, more methadone would be pumped out and, consequently, higher methadone doses may be required to maintain the therapeutic level of methadone.

Another concern for the variant genotypes of P-gp in patients under methadone maintenance therapy is the treatment of HIV infection, which is a common complication in these patients. Protease inhibitors are the key component in the Highly Active AntiRetroviral Treatment (HAART) for HIV treatment. It has been demonstrated that the widely used protease inhibitors, such as indinavir, lopinavir, nelfinavir, ritonavir and atazanavir, are substrates of P-gp [23], [32]–[34]. Moreover, patients with 3435TT genotypes were associated with lower plasma concentrations of nelfinavir and atazanavir [46], [47] while patients with wild-type P-gp tended to display higher plasma concentrations of atazanavir and ritonavir [48]. The drug-drug interactions between commonly used protease inhibitors and methadone were extensively reviewed and it has been suggested that aware of the potential interactions may help patients from experiencing drug craving, withdrawal or intoxication [49]. From the present study, methadone was shown to inhibit wild-type P-gp most efficiently in the tested genotypes; therefore, patients with wild-type P-gp may suffer from adverse drug reactions of high atazanavir and ritonavir plasma concentrations due to concomitantly used methadone. The haplotype frequencies of the wild-type P-gp (1236C-2677G-3435C) in Caucasian and Chinese populations were 0.2 and 0.15, respectively [10], [11]. Thus, with the high haplotype frequencies, the inhibition effect of methadone on P-gp may lead to drug-drug interaction in clinical practice.

The strength of the present study may include 1) unveiling the kinetic mechanism of methadone-P-gp interaction, and 2) providing evidence that variants of the *ABCB1* gene may affect both the potency and kinetics of this interaction. Furthermore, methadone was demonstrated to be a P-gp ATPase stimulator under clinical plasma concentration. Although the present study revealed the important methadone-P-gp interaction, lack of clinical data to support the hypothesis of drug-drug interaction is the major limitation. Therefore, further clinical study is needed.

In conclusion, the present study demonstrated that methadone is a human P-gp inhibitor and showed distinctive mechanism in variant-type human P-gp. These results provide basic evidence for drug-drug interactions, which physicians should take into account when combination therapy is applied. Nonetheless, further clinical studies are warranted to confirm the impact of the P-gp mediated drug-drug interactions as well as the unique effect of the variant-type P-gp.
